# Systematic comparison of single-chain Fv antibody-fusion toxin constructs containing Pseudomonas Exotoxin A or saporin produced in different microbial expression systems

**DOI:** 10.1186/s12934-015-0202-z

**Published:** 2015-02-13

**Authors:** Pietro Della Cristina, Monica Castagna, Alessio Lombardi, Erika Barison, Giovanni Tagliabue, Aldo Ceriotti, Ilias Koutris, Luana Di Leandro, Francesco Giansanti, Riccardo Vago, Rodolfo Ippoliti, Sopsamorn U Flavell, David J Flavell, Marco Colombatti, Maria Serena Fabbrini

**Affiliations:** Department of Pathology and Diagnostics, University of Verona, Verona, Italy; Istituto Biologia e Biotecnologia Agraria, CNR, Milan, Italy; Department of Life, Health and Environmental Sciences, University of L’Aquila, L’Aquila, Italy; The Simon Flavell Leukaemia Research Laboratory, (Leukaemia Busters), Southampton General Hospital, Southampton, UK; Istituto Nazionale di Genetica Molecolare-INGM, Milan, Italy

**Keywords:** Recombinant immunotoxins, Anti-CD22, Pseudomonas exotoxin A, Saporin, Bacterial/eukaryotic expression systems

## Abstract

**Background:**

Antibodies raised against selected antigens over-expressed at the cell surface of malignant cells have been chemically conjugated to protein toxin domains to obtain immunotoxins (ITs) able to selectively kill cancer cells. Since latest generation immunotoxins are composed of a toxic domain genetically fused to antibody fragment(s) which confer on the IT target selective specificity, we rescued from the hydridoma 4KB128, a recombinant single-chain variable fragment (scFv) targeting CD22, a marker antigen expressed by B-lineage leukaemias and lymphomas. We constructed several ITs using two enzymatic toxins both able to block protein translation, one of bacterial origin (a truncated version of *Pseudomonas* exotoxin A, PE40) endowed with EF-2 ADP-ribosylation activity, the other being the plant ribosome-inactivating protein saporin, able to specifically depurinate 23/26/28S ribosomal RNA. PE40 was selected because it has been widely used for the construction of recombinant ITs that have already undergone evaluation in clinical trials. Saporin has also been evaluated clinically and has recently been expressed successfully at high levels in a *Pichia pastoris* expression system. The aim of the present study was to evaluate optimal microbial expression of various IT formats.

**Results:**

An anti-CD22 scFv termed 4KB was obtained which showed the expected binding activity which was also internalized by CD22^+^ target cells and was also competed for by the parental monoclonal CD22 antibody. Several fusion constructs were designed and expressed either in *E. coli* or in *Pichia pastoris* and the resulting fusion proteins affinity-purified. Protein synthesis inhibition assays were performed on CD22^+^ human Daudi cells and showed that the selected ITs were active, having IC_50_ values (concentration inhibiting protein synthesis by 50% relative to controls) in the nanomolar range.

**Conclusions:**

We undertook a systematic comparison between the performance of the different fusion constructs, with respect to yields in *E. coli* or *P. pastoris* expression systems and also with regard to each constructs specific killing efficacy. Our results confirm that *E. coli* is the system of choice for the expression of recombinant fusion toxins of bacterial origin whereas we further demonstrate that saporin-based ITs are best expressed and recovered from *P. pastoris* cultures after yeast codon-usage optimization.

**Electronic supplementary material:**

The online version of this article (doi:10.1186/s12934-015-0202-z) contains supplementary material, which is available to authorized users.

## Background

Over a century ago Paul Ehrlich formulated a new idea in medicine, the “magic bullet” concept, in which a drug would be selectively directed against a pathogen/cellular target and which would therefore be innocuous to the surrounding healthy tissues. This concept was later realized by the discovery of monoclonal antibodies, providing us with molecules endowed with antigen-specific binding capability [1] thus opening the way for the first generation of immunotoxins (ITs) constructed with whole antibodies conjugated to chemically modified toxic domains. These first generation ITs were created by cross-linking monoclonal antibodies directed against marker antigens overexpressed on the tumor cell surface to toxin protein domains of choice, derived either from plants like saporin or ricin A chain or as *Diphtheria* and *Pseudomonas* toxin domains, from bacteria. However, these type of ITs possessed several weaknesses as follows: 1) heterogeneity among different batch preparations, 2) high immunogenicity and 3) safety issues and high costs for their production under GMP conditions [2].

This led to the development of a new generation of recombinant chimeric molecules (for a review *see* [3-5]) which are not only easier to manipulate but which also yield ITs endowed with consistent physico-chemical properties. In particular, toxic enzymatic sequences can be directly genetically fused to sequences encoding the selected targeting domains (e.g. hormones, growth factors, antibody portions, including single-chain variable fragments (scFv)). Additionally, toxin molecules can be engineered to delete unwanted native cell-binding domains while retaining those domains involved in cell membrane translocating activity. Targeting domains might also be further modified to enhance their cellular specificity, binding affinity, etc.

Neoplastic B-cells arising in hematopoietic malignancies frequently express at their surface the CD19 and CD22 differentiation antigens. CD22 is not expressed by any other normal tissue being restricted to only normal and malignant B-cells making this a good candidate target molecule for antibody-targeted therapies. A combination of anti-CD19, −CD22, and -CD38-saporin ITs (3BIT cocktail) has been shown previously to cure severe combined immunodeficient mice xenografted with the human B-cell lymphoma cell line Ramos, resulting in 100% disease-free survivors at 300 days [6]. Several first generation anti-CD22 ITs have been described in the past some chemically conjugated to plant deglycosylated ricin A-chain [7] and others to *Pseudomonas* Exotoxin A (PEA) that have yielded encouraging results *in vivo* in animal models and in clinical trials in humans [8]. However, due to some of the above-mentioned limitations, development of fully recombinant anti-CD22 ITs is highly desirable for therapeutic use in humans. BL22 is a fusion protein derived from the parental anti-CD22 RFB4 monoclonal antibody formed between an anti-CD22 disulfide-stabilized antibody fragment (dsFv) and a shorter version of bacterial PEA termed PE38. In 2001 results were reported of complete remissions in a phase I trial for hairy cell leukemia [9]. A next generation IT (High affinity BL22) molecule, HA22 [3,10], incorporated a three amino acid change in the antibody fragment to increase the binding affinity for the target CD22 molecule and is currently under clinical evaluation by NIH.

Single-chain fragment variable antibody fragments (scFv) are recombinant molecules which can be derived from phage display libraries [11] or alternatively from hybridomas secreting whole murine antibodies by RT-PCR amplification of the variable antibody domain sequences. Although of murine origin, the scFv represent a less immunogenic portion of the antibody molecule. Humanization of murine scFv would further reduce their immunogenicity and help to prevent neutralizing or damaging immune responses following repeated administration to patients. Avoiding an immune response against the toxic moiety is more problematical, but strategies have been developed to minimize this and allow repeated administrations *in vivo*. For example, PE38, a recombinant version of *Pseudomonas* Exotoxin A could be de-immunized by deletions/substitution of the main immunogenic residues [12-14]. Alternatively, fusion toxins may be engineered using a weakly immunogenic [15,16]; (Flavell et al., unpublished observations) toxin such as saporin from *Saponaria officinalis*. Thus different toxic portions could easily be swapped into chimeric recombinant constructs, retaining the same targeting domain, firstly allowing the immunological response against the toxic moiety to be reduced and secondly to provide the opportunity to swap in a different toxin domain whilst retaining the same target antigen specificity.

In the present study, we compared different constructs containing the same recombinant anti-CD22 scFv fused to two different toxin domains: PE40, a truncated version of *Pseudomonas* exotoxin A, or saporin. Both were expressed either in prokaryotic (i.e. *E. coli*, already described for PE40-based IT [17]) or eukaryotic (i.e. *Pichia pastoris*, already described for saporin [16]) microbial hosts, in order to set-up the most appropriate conditions for the rapid development of new anti-CD22 recombinant ITs.

We made fusion proteins between an scFV derived from a previously described anti-CD22 murine IgG_1_ antibody (4KB128, [18]) which formerly demonstrated excellent targeting properties as a carrier of native seed-derived saporin against a human B-cell lymphoma cell line [6] and full length saporin or PE40 as the toxin moiety.

Overall our results demonstrate that IT containing a toxin moiety of bacterial origin are better expressed in the *E. coli* host, while saporin-based ITs are best expressed in the *P. pastoris* system. The potency of the resulting IT molecules obtained was comparable, with the PE40-based IT showing a 5-fold higher cytotoxic activity in some experiments.

## Results and discussion

### Rationale for the design of experiments

To date, bacterial and yeast host cells have been used to produce RIPs or RIP-based ITs [19,20]. One common problem faced during the production of recombinant RIPs resides in their intrinsic toxicity toward the host ribosomes. Toxin expression can be rapidly achieved in bacteria and tightly regulated by employing specific E. coli strains, to obtain satisfactory yields [21,22], but in some cases the protein may accumulate inside the cell as an insoluble fraction from which fully active RIP is not easily recoverable. Endotoxin contamination together with inefficient folding of certain secretory targeting domains appear to be the main disadvantages of the bacterial expression systems and this has prompted the more recent development of eukaryotic expression systems. The methylotrophic yeast *Pichia pastoris* has been demonstrated to be a suitable platform for the expression of recombinant proteins, allowing protein post-translation modifications and a several-fold yield improvement in product [23]. Recombinant DT-based IT fusions has been successfully expressed in *P. pastoris*, in the GS115 strain that was found to be particularly tolerant to this bacterial toxin [24]. Toxicity was most likely prevented through rapid and efficient secretion of the toxin into the culture medium, allowing for easier downstream purification and production scale up. On the other hand, yeast expression systems for the production of toxins takes longer than for bacterial expression systems and concomitantly secreted or membrane-bound enzymes can proteolytically cleave the expressed recombinant proteins. In this regard the two toxic domains we used in the production of fusion ITs match some of the requirements, since Pseudomonas exotoxin A has been successfully used to construct recombinant ITs expressed in *E. coli* [17] in the truncated PE38 version, easily recovered from inclusion bodies, while saporin has been expressed as both free toxin or fusion IT [16] by our group in *Pichia pastoris* and is easily purified from the culture medium. In the latter case we noticed a strong influence of the antibody moiety on the stability and intracellular processing of the recombinant IT in the eukaryotic system. Taking these aspects into consideration we decided to systematically compare anti-CD22 based scFV fused with the two toxins in the prokaryotic (*E. coli*) and eukaryotic (*Pichia pastoris*) expression systems.

### Selection of the anti-CD22 single chain variable regions and characterization of 4KB scFv constructs expressed in *E. coli*

**Figure 1:**
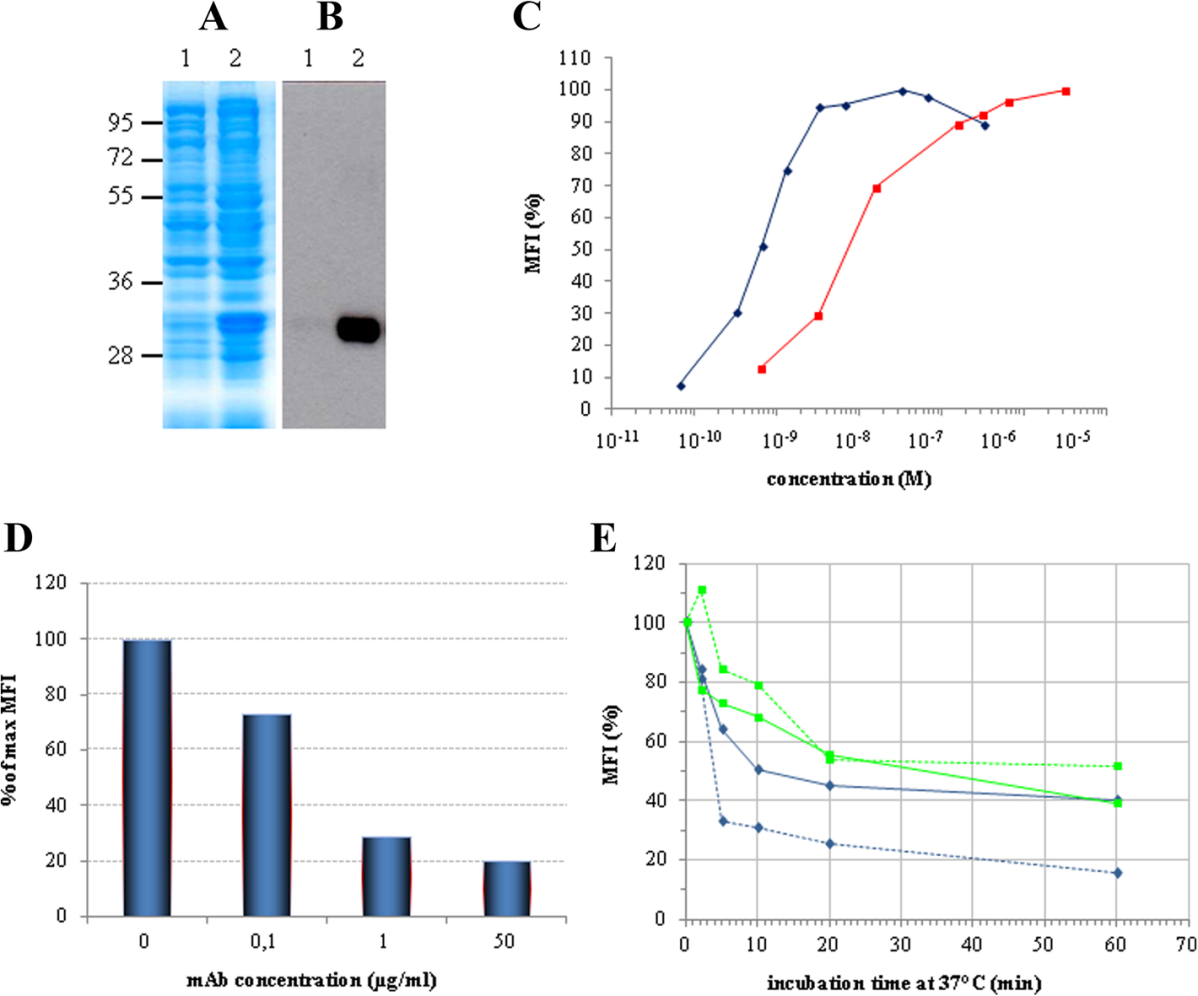
**Expression & characterization of the 4KB scFv.** Total lysate of non-induced (*lane 1*) and IPTG-induced (*lane 2*) *E. coli* BL21(DE3) pLys transformed with pET20b(+)4KBscFv were loaded and the expression of the recombinant protein was detected by **(A)** Coomassie blue staining or **(B)** Western blot analysis with anti-His antibody. **(C)** The binding activity of 4KB scFv (red squares) was compared with that of 4KB128 mAb (blue diamonds) by flow-cytometric analysis on Daudi cells incubated at 4°C using increasing amounts of purified 4KB128 mAb or 4KB scFv. **(D)** The binding of 4KB scFv (50 μg/ml) on Daudi cells is competitively inhibited by increasing concentrations of the parental anti-CD22 mAb pre-incubated with the cells. The scFv-associated fluorescence without competing mAb pre-incubation is taken as the maximal reference MFI. **(E)** Internalization and stability of the anti-CD22 mAb compared to 4KB scFv. Ramos (light blue) and Daudi (green) cells were stained at 4°C with 30 μg/ml 4KB scFv (continuous line) or 10 μg/ml mAb (dashed line) and subsequently incubated at 37°C for the indicated times, as described in Methods. Red lines indicate the MFI obtained by staining Daudi cells with the scFv (continuous line) and mAb (dashed line) previously incubated at 37°C for the same time lengths as for the internalization experiment. MFI values are plotted as percentage relative to the fluorescence obtained for samples kept on ice.

A set of primers (forward and reverse, see Additional file 1: Table S1) was used to amplify the heavy (V_H_) and light (V_L_) variable antibody domains from hybridoma cells on reverse transcribed anti-CD22 hybridoma mRNA. We obtained the two selected variable domains that were subsequently assembled, as described in the Methods section (see below), inserting a (G_4_S)_3_ (one letter amino acid code) peptide linker joining the two polypeptides. This first DNA construct was subcloned, sequenced and then expressed in *E. coli* BL21(DE3)pLysS cells with a C-terminal hexahistidine tag to allow easy nickel-affinity purification. The level of scFv expression in BL21(DE3)pLysS was first assessed in small-scale cultures. Following IPTG induction, an overexpressed band with an expected size of approximately 30 kDa was detected in Coomassie blue-stained SDS-PAGE gels (Figure 1A, lane 2) which was also specifically recognized by an anti-histidine antibody in Western blotting (Figure 1B, lane 2).

The 4KB scFv was next expressed in higher amounts, being found in inclusion bodies from where it was extracted after protein denaturation in a urea-containing buffer followed by purification by nickel-affinity chromatography (see, Methods section). Attempts to refold the purified proteins did not allow for the full recovery of the purified denatured molecules, which were largely lost through precipitation during this procedure, presumably due to incorrect folding, as the denaturing agent was gradually removed. Despite these problems, the final yield was approximately 4 mg of purified 4KB scFv from a 1 l of *E. coli* fermentation liquor.

### Characterization of the binding of the parental anti-CD22 monoclonal antibody and derived scFv

Before generation of anti-CD22 ITs, the binding properties of the parental IgG1 mAb and the derived scFv to the native cellular antigen were confirmed by flow cytometry on CD22^+^ lymphoid cell lines. As shown in Figure 1C, a Mean Fluorescence Intensity (MFI) curve was obtained following staining CD22 expressing cells with increasing concentrations of mAb (blue line) or scFv (red line). The expected sigmoid shaped curve was obtained on Daudi cells (CD22^+^) but as expected binding was not seen on two CD22 negative T-lymphoblastoid cell lines (H9 and HSB-2) as negative controls (data not shown). On CD22^+^ Daudi cells the MFI-plateau above 3 nM of mAb, whilst 4KB scFv showed a 10-fold reduced affinity to the same cellular target in comparison to the native bivalent mAb.

The specificity of the molecular target recognized by the anti-CD22 scFv was also confirmed by analyzing 4KB scFv binding on CD22-expressing cells, in a competition assay with increasing concentrations of the parental mAb. The scFv-associated fluorescence decreased in a dose-dependent manner as the amount of anti-CD22 mAb used to pre-stain cells was increased (Figure 1D).

Finally, the avidity of the specific binding of 4KB scFv to the recombinant extracellular domain of CD22 was determined using Biacore. The dissociation constant (*K*_*d*_) of the interaction between 4KB scFv and recombinant CD22 target antigen was assessed using Surface Plasmon Resonance technology. The resulting *K*_*d*_ (k_*off*_/k_*on*_) evaluated was 5.1 × 10^-8^ M for the scFv (data not shown), a value consistent with a *K*_*d*_ of 2.5 × 10^-9^ M previously determined for the parental 4KB128 monoclonal antibody (our unpublished observations), supporting the likely suitability of 4KB scFv for IT constructions.

To ensure that our scFv represented a suitable delivery vehicle for the design of an immunotoxin, the internalization capability of the antibody fragment was also investigated by flow cytometry, following binding to CD22 expressed on the surface of target Daudi and Ramos cells. By plotting the fluorescence associated with residual surface-bound scFv against incubation time at 37°C, a rapid fall in extracellular staining was observed, indicating rapid endocytosis of bound antibody, particularly in Ramos cells (Figure 1E). It is apparent that the endocytosis trend almost overlaps with the native bivalent mAb and univalent 4KB scFv, indicating that the targeted site(s), rather than the valency of the binding antibody, is the critical factor in determining the efficiency of uptake. Both antibodies preserved their binding capability (binding at 4°C) of the two target cell lines even after a prolonged pre-incubation at 37°C (data not shown), ruling out the possibility that decrease in MFI may have been due to intrinsic antibody instability, degradation, dissociation or antigen shedding following incubation at 37°C.

### Production and characterization of the 4KB derived fusion constructs expressed in *E. coli*

**Figure 2:**
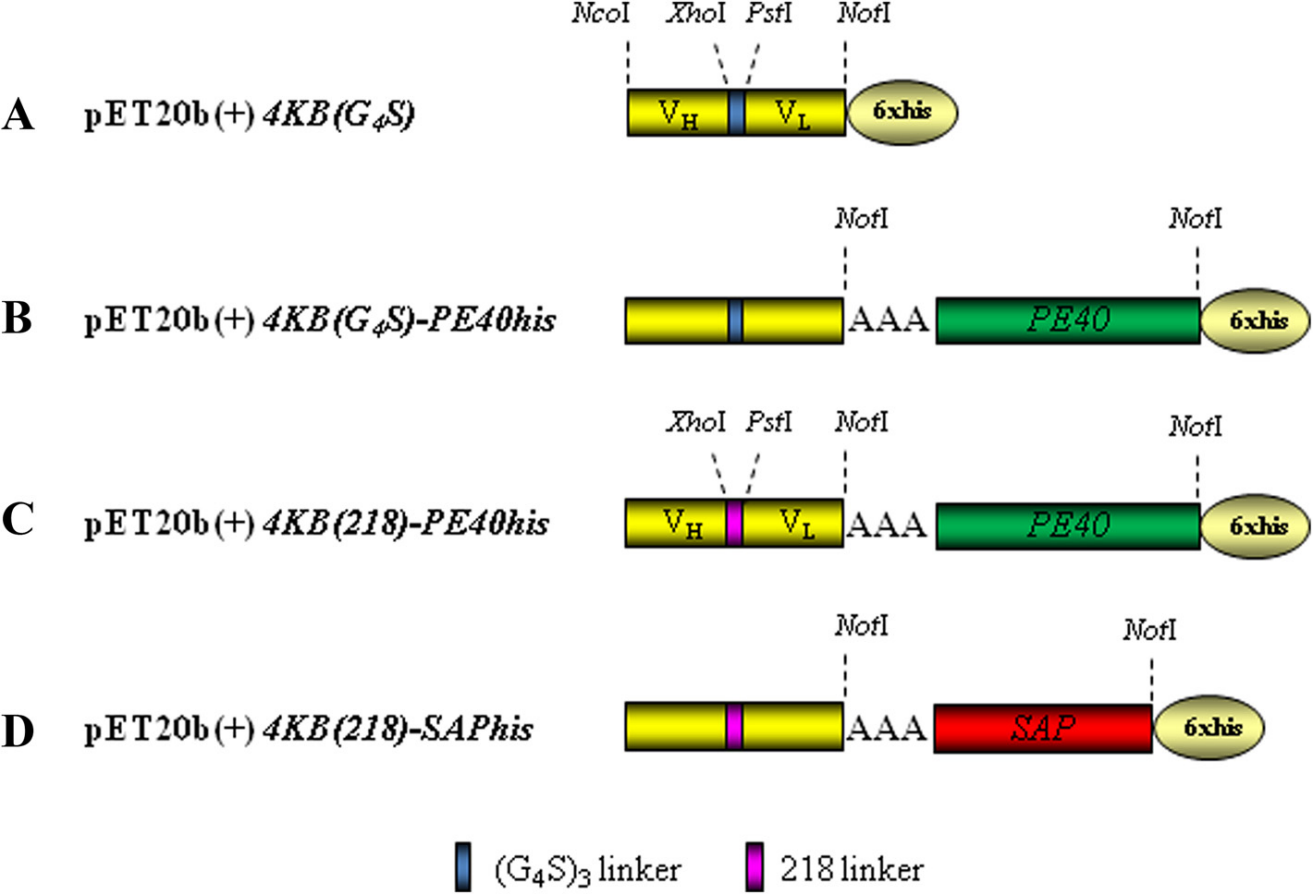
**Constructs for the expression of toxin-based fusions in** ***E. coli.*** Schematic representation of 4KBscFv **(A)**, PE **(B and C)** and saporin **(D)**-derived constructs. Restriction enzyme sites used for the cloning strategy are also shown (for details, see text under Methods section). Sequence of the 218 linker (218 L) in fuchsia color is: GSTSGSGKPGSGEGSTKG (amino acid one letter code).

**Figure 3:**
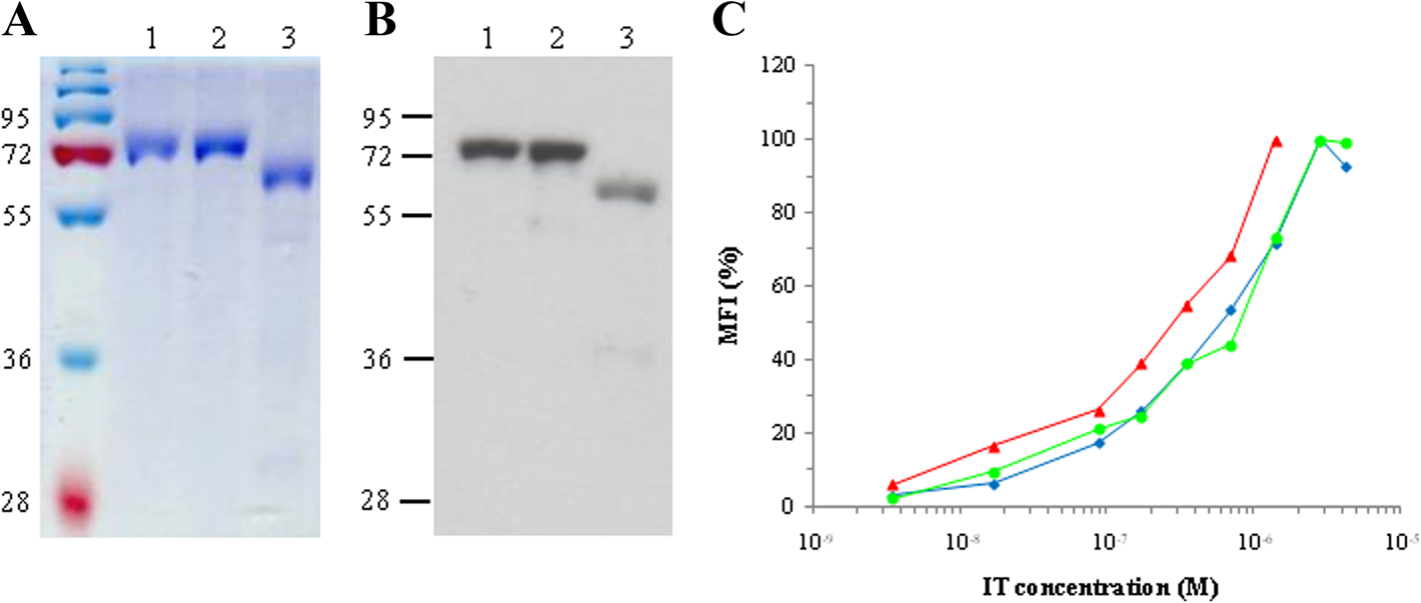
**Characterization of recombinant ITs expressed in** ***E. coli*** **purified by IMAC.** **(A)** Coomassie staining and **(B)** Western blot with anti-His antibody of purified 4KB-PE40 in *lane 1*, 4KB(218)-PE40 in *lane 2* and 4KB(218)-SAP in *lane 3*. **(C)** Comparison of the binding characteristics of 4KB-PE40 (blue diamonds), 4KB(218)-PE40 (green circles) and 4KB(218)-SAP (red triangles) analyzed by flow-cytometry using Daudi cells incubated at 4°C with increasing concentrations of each IT.

The nucleotide sequence coding for the PE40 truncated version of *Pseudomonas* exotoxin A was fused to the 3’-end of the 4KB scFv, generating a chimeric immunotoxin encoded within the pET20b(+) vector (Figure 2B). The C-terminal hexahistidine tag was exploited for purification and analytical purposes. The small-scale expression of the recombinant IT (rIT) in BL21(DE3)pLysS *E. coli* yielded an induced protein of approximately 70 kDa, consistent with the expected size for a fusion between the scFv (30 kDa) and PE40 (40 kDa) (Figure 3A and B).

This preliminary induction assay showed that, unlike the scFv, the derived rIT could be expressed as a single molecular species, possibly retaining the N-terminal signal peptide for periplasmic sorting. Although its level of synthesis seemed to be appropriately lower than that of the scFv alone, this did not prevent accumulation of the chimeric protein exclusively in inclusion bodies, as no detectable rIT could be recovered in soluble form(s) either in the cytoplasmic or in the periplasmic compartments (data not shown), indicating a certain propensity of the fusion toxin to aggregate, presumably due to the presence of the anti-CD22 recombinant scFv domain.

A larger culture was thereafter grown, induced and processed to extract the chimeric protein from inclusion bodies which was then purified and refolded, as described in Methods. This procedure allowed us to recover approximately 3 mg/L of rIT from induced bacterial culture, a yield consistent with those previously reported for other recombinant ITs that include truncated versions of PEA [25]. A distinguishing feature of our rIT, as compared to the scFv polypeptide alone, was a negligible loss of the rIT during the renaturation step. We calculated that approximately 80% of the denatured recombinant protein eluted by IMAC was recoverable after the refolding procedure.

**Figure 4:**
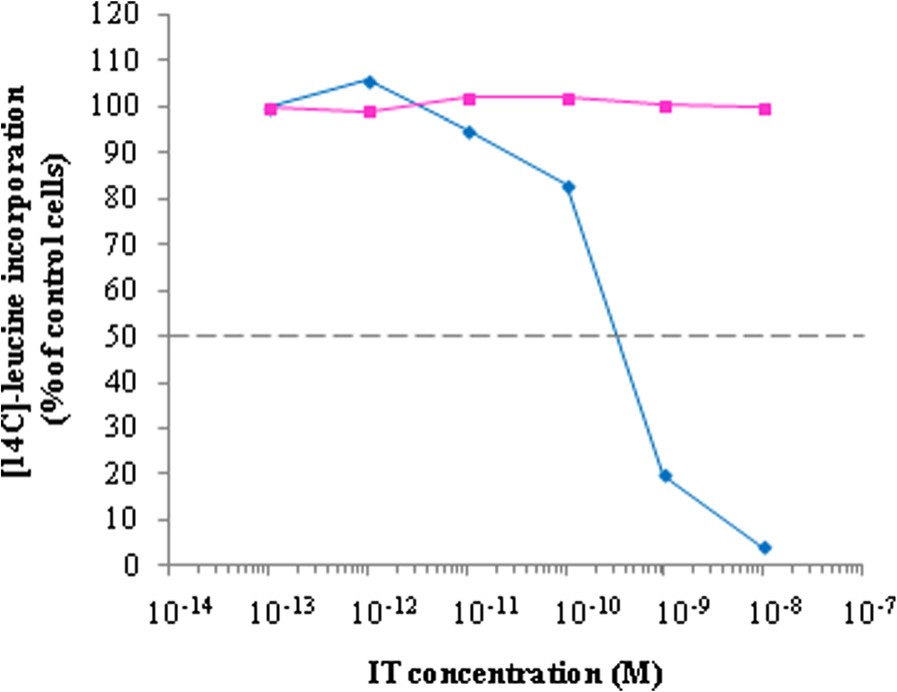
**Characterization of 4KB-PE40 IT immunospecificity for CD22 expressed on Daudi cells.** The cytotoxic assay was performed incubating Daudi cells for 72 hours with increasing concentrations of 4KB-PE40 in the presence (pink squares) or absence (blue diamonds) of a fixed concentration of the corresponding parental 4KB128 monoclonal antibody. Inhibition of protein synthesis is expressed as percentage of [^14^C]-leucine incorporation compared to the control samples (untreated cells).

4KB-PE40 has a good binding capacity as demonstrated by flow cytometry on Daudi cells (Figure 3C). Furthermore, to assess the biological activity of our first fusion construct we performed protein synthesis dose–response assays which demonstrated a cytotoxic activity of 4KB-PE40 on Daudi cells with an IC_50_ of approximately 0.3 nM (Figure 4). The cytotoxicity observed was dependent on the presence of the anti-CD22 scFv domain fused to PE40 since the toxin alone or the scFv alone were substantially less effective against Daudi cells, while in turn the cytotoxicity of the rIT towards CD22 negative cell lines was, as expected, significantly less (Table 1). Additional evidence of the immunospecificity of our rIT for CD22 as the target antigen is further supported by the observation that co-incubation with an excess of whole monoclonal parental antibody abolished the cytotoxicity of rIT, indicating displacement of the rIT by the competing whole antibody (Figure 4).

**Table 1:** **Comparison of concentrations of the 4KB-PE40 IT, PE or the scFv alone inhibiting protein synthesis by 50% of control values (IC**_**50**_ **)**

	**Daudi**	**Ramos**	**HSB-2**	**H9**
	**IC**_**50**_			
IT	7 nM	4 nM	>300 nM	>300 nM
PE	200-300 nM	>750 nM	60 nM	>750 nM
scFV	>3200 nM	>3200 nM	>3200 nM	>3200 nM

CD22^+^ cell lines Daudi and Ramos or CD22^−^ lines HSB-2 and H9 were exposed for 48 h to the 4KB scFv-derived immunotoxin (IT) or to native PE exotoxin A (PE) or 4KB antibody fragment alone (scFv) and cytotoxicity was evaluated by protein synthesis inhibition assay as described in the Methods section.

The sequence coding for PE40 was also sub-cloned at the C-terminus of a different 4KB scFv format in which the V_H_ and the V_L_ domains were joined through the 218 linker (Figure 2C), a more flexible and hydrophilic sequence [26].

The purified 4KB(218)-PE40 fusion protein showed chemical and physical properties similar to that of 4KB-PE40. The recombinant IT had a molecular mass of approximately 70 kDa and was recognized by the anti-His antibody in Western blotting (Figure 3A-B, lane 2). Additionally, the levels of synthesis and the final yields of the latter fusion protein were also comparable to those of the first rIT made with the (G_4_S)_3_ linker.

In parallel experiments, we utilized the latter anti-CD22 scFv to deliver the 30 kDa plant-derived toxin RIP saporin. Since a more flexible and hydrophilic linker may be advantageous for the construction of a rITs, we decided to link the sequence coding for a plant saporin isoform [27] to the 4KB(218) scFv version and the latter rIT was also expressed in bacteria and purified, as described for the PEA-based recombinant proteins (see Methods). However, in the case of rIT containing a saporin domain we observed a lower level of rIT synthesis than that observed for PE40 containing rIT in *E. coli* following IPTG induction. This phenomenon was apparently not dependent on possible host auto-intoxication effects observed during saporin expression in several hosts [28], since the *E. coli* growth curve of the bacterial transformant strain was not influenced by the expression of the fusion protein (data not shown).

Nevertheless, around 4 mg/L of this saporin fusion protein could be extracted from inclusion bodies but more than 90% was lost during the renaturation process due to aggregation and concomitant precipitation caused by what we presume must be due to the instability of this particular IT construct. Indeed it has been shown previously that saporin and fusion proteins incorporating this RIP have a low propensity to refold after urea denaturation procedures (D. Lappi, personal communication).

The binding characteristics of the different recombinant ITs produced by the bacterial expression system were compared by flow cytometry as described in Methods. As shown in Figure 3C the data demonstrate overlapping binding curves on Daudi cells.

**Figure 5:**
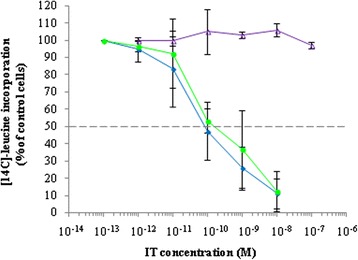
**Cytotoxicity of 4KB128-derived rITs for CD22** ^**+**^ **Daudi cells.** Protein synthesis inhibition assay on Daudi cells exposed for 72 hours to increasing concentrations of 4KB-PE40 (blue diamonds), 4KB(218)-PE40 (green circles) or 4KB(218)-SAP (violet triangles). Protein synthesis inhibition is expressed as a percentage of [^14^C]-leucine incorporation compared to untreated control cells. Error bars represent standard deviations from the mean of triplicate samples.

**Figure 6:**
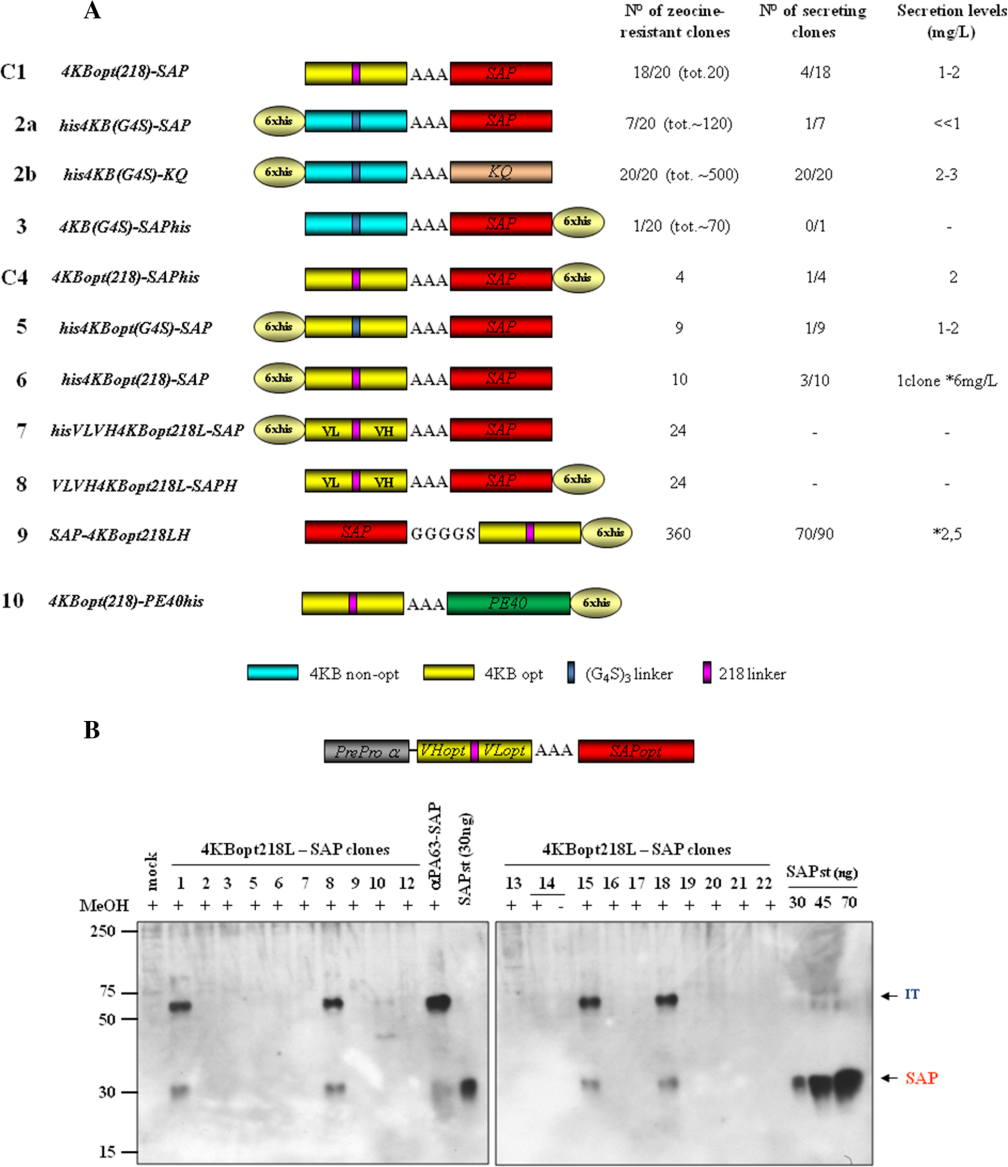
**4KB-SAP and 4KB-PE40 fusions expressed in** ***Pichia pastoris*** **G115 (** ***his4*** **). (A)** Schematic representation of saporin- (C1-9) or PE-based (10) -4KB128-derived fusion constructs. C1: *Pichia*-fully optimized Construct 1 expressing clone. **(B)** Western blot analysis of 4KBopt218L-SAP clones. Secretion yields were estimated at 1-2 mg/L and compared to induced mock or positive control anti-PA63scFv-SAP expresser clones.

Next, rITs produced in bacteria were tested in a protein synthesis inhibition assay on Daudi cells (Figure 5). 4KB-PE40 (green) and 4KB(218)-PE40 (blue) showed very similar cytotoxic activities with an IC_50_ of approximately 0.1 nM, while unexpectedly, the 4KB(218)-SAP produced in *E. coli* (violet) failed to show any cytototoxicity, we presume due to IT instability problems, as alluded to above. We did not assay the 4KB(G_4_S)_3_-SAP construct, since parallel experiments performed in *P. pastoris* demonstrated that this construct was incapable of giving rise to inducible clones in the *P. pastoris* expression system (see Figure 6).

Overall, these data confirm that rITs formed by PE40 fused to the anti-CD22 scFv joined by different linker peptides can be successfully produced and purified in *E.coli* and, most importantly, are biologically active. In contrast, a similar construct based on a saporin toxin domain was not properly expressed in bacteria and the renatured purified rIT molecules therefore failed to intoxicate CD22^+^ target cells.

### Selection of the 4KB derived, best-suited fusion constructs expressed in *P. pastoris*

Saporin and a number of recombinant fusion proteins have been previously expressed with some success in *E. coli* [4]. However, eukaryotic hosts would seem much more suitable for expression of saporin chimaeras [29], as we recently demonstrated by exploiting the microbial eukaryotic host *Pichia pastoris* as an expression platform [30]. Having observed the production of aggregation-prone product(s) during expression of our anti-CD22 PE40 IT in *E. coli*, and having obtained low, non- functional amounts of this saporin-based IT in bacteria, we decided to compare the expression of companion saporin-based ITs in *P. pastoris*.

With this aim, we prepared a panel of constructs (see, Figure 6A) fusing the sequences coding for the anti-CD22 V_H_ and V_L_ domains alternatively connected by using (G_4_S)_3_ or 218 linkers, as described for 4KB-PE40, to a saporin yeast-optimized sequence [30] either carrying an N- or C-terminal hexahistidine tag.

The first attempts to replate zeocine-resistant transformed clones and induce fusion protein expression were unsuccessful as we obtained only a very low number of transformants, in some instances as few as only one or two transformant zeocine-resistant clones, which were incapable of expression induction (Figure 6A, for examples see schemes for constructs 2a and 3).

As a control, *Pichia* cells transformed with an enzymatically inactive saporin mutant construct termed 4KB-SAPKQ (named KQ because a Lysine K and a Glutamine Q residue were introduced at the saporin catalytic site) yielded plates with the expected number of several hundred viable growing colonies (Figure 6A, see scheme for construct 2b) all of which were zeocine-resistant and all of which could be induced to express, on a small-scale, up to 2 mg/L of the fusion protein containing inactive mutant KQ saporin. This observation suggests that one likely reason for the unsuccessful expressions of IT was the high toxicity of the enzymatically fully active saporin domain towards host *Pichia* cells. Similar effects have also been previously reported during the expression of wild type saporin or chimaeras containing saporin fused with the Amino-Terminal Fragment of human urokinase (ATF-saporin) in different host cells, including *P. pastoris* [28].

Expression of the 4KB scFv construct alone yielded the expected number of secreting clones, and these clones were further analyzed following a medium scale induction (see Additional file 2: Figure S1) which all gave a good secretory yield, further supporting the notion that the low number of transformants we obtained for rIT constructs was in all likelihood due to intoxication of the host by fully active saporin.

Codon-optimization has already been shown to markedly decrease the toxicity issues associated with saporin expression in *P. pastoris* and also found to be essential for obtaining clones that expressed high levels of active saporin [30]. For these reasons, we decided to design a yeast codon-optimised anti-CD22 sequence for fusion to the N-terminus of mature saporin through a trialanine linker, which has previously been successfully used for recombinant ATF-saporin constructs [29] (Figure 6A).

A synthetic optimized gene was therefore assembled as described in the Methods section in which a yeast codon-optimized sequence coding for saporin [30] fused with different versions of the scFv with either a (G_4_S)_3_ linker, not sequence optimized (colour coded turquoise in Figure 6A) or the 218 linker (colour coded purple) joining the V_H_ and V_L_ codon-optimized variable chains (colour coded yellow in Figure 6A) for expression in *P. pastoris*. Among all the constructs obtained, constructs termed C1 and C4 were then analyzed further as described below.

Codon-optimization of the scFv domain appears to be important to enable an increase in the potential number of secreting clones that are capable of achieving a minimum of 1–2 mg/L of fusion protein production. In the supplementary figures we show some additional data for constructs 6 and 9 that gave rise to expresser clones in medium scale inductions that reached values as high as 5–10 mg/L. However, neither of these had any demonstrable saporin catalytic activity even when they were selected among a much larger number of transformants, directly on plates (see Additional files 3, 4, 5 and 6: Figures S2-S5). Indeed, Construct 9 which has the saporin C-terminus blocked by a G_4_S linker peptide that joins the toxin to the scFv domain, showed the largest number of transformant (360 clones) but no enzymatic activity was detectable when the purified fusion protein was assayed (data not shown).

Appending extensions at the C-terminus has previously been reported to cause inactivation of saporin (Sap-VSAV, single letter aminoacid code) assayed by an *in vitro* cell-free inhibition assay, but enzymatic activity was “activated” once this molecule was used against whole viable cells [21] suggesting that a proteolytic activation step takes place either extra- or intracellularly.

Finally, constructs 5 and 6 expressed with an hexahistidine tag appended at the N-terminus of the scFv were not recognized by an anti-his polyclonal antibody (Additional file 6: Figure S5), suggesting that proteolytic removal of this tag may have taken place, as shown for the PEA fusion as described below.

Since it is known that a gelonin-based IT (having a VL domain connected to the VH antibody domain via the 18-amino acid 218-flexible linker GSTSGSGKPGSGEGSTKG, amino acid one letter code) shows enhanced resistance to proteolysis and reduced aggregation properties of scFvs when expressed in bacterial systems [26,19], we decided to make two constructs (constructs 7 and 8 in Figure 6A) that were designed with a reversed VL-VH configuration, in contrast to all the other constructs.

Among alternate construct configurations that we also explored, the hexahistidine tag appended at N-terminus in the IT (Figure 6A, contructs 5 and 6) or the saporin domain cloned at N-terminus of the scFv (Figure 6A, construct 9) gave rise to fusion polypeptide produced in medium scale with considerable yields (see Additional files 3, 4 and 5: Figures S2-S4), but when they were purified and tested on Daudi cells, no cytotoxic activity was detected (data not shown). Finally, when VH-VL orientation constructs were prepared (Figure 6A, constructs 7 and 8) in the hope of increasing the scFv stability/flexibility or its affinity towards the target antigen, as previously demonstrated by others [31], no expression was obtained. (see Additional files 3, 4 and 5: Figures S2-S4).

Overall, we may draw the following conclusions from the data we obtained with the VH-VL configurations examined so far. Our results indicate that 4KB scFv behaves as a poor secretory domain, prone to aggregation (found in inclusion bodies in bacteria) and undergoes misfolding which may explain why transformation of fusion constructs containing an active saporin domain resulted in a very few transformants: if the misfolded polypetides were retro-translocated to the cytosol for degradation by the ER-associated degradation pathways, saporin would escape segregation in the endoplasmic reticulum being active against cytosolic ribosomes. Consistently, secretion levels of the KQ control fusion protein (contruct 2b, Figure 6) were also extremely low, at least 10 times lower than when saporin KQ is expressed alone in GS115(*his4*) [30]. This would suggest that when this scFv domain is fused even to a good secretory protein it has direct detrimental effects on the overall expression/secretion levels.

### An example of saporin-based CD22 immunotoxin expressed in *Pichia pastoris*

Notwithstanding the major problems of expression, among the *Pichia* zeocine–resistant transformants obtained, twenty independent clones were available for screening for inducible expression. The best expressing clones were selected following screening in 5–10 mL, in small-scale inductions [30]. Expression yields for the ITs ranged between 1 and 2 mg/L (Figure 6B). We next undertook medium-scale preparations starting at a turbidity of 10 OD/mL which were prepared and induced for 48 h as described previously (see S1 as a representative example and [30]). Collected media were concentrated and dialysed before purification. We used affinity chromatography to purify His-tagged fusion proteins or as an alternative cation exchange chromatography that exploits saporin’s extremely high PI [4,28,2].

**Figure 7:**
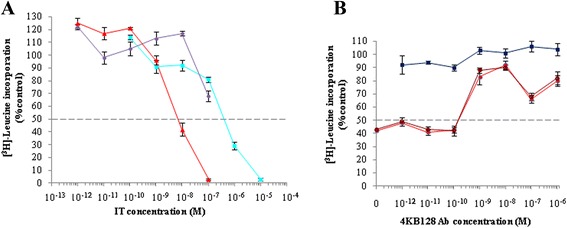
**Cytotoxicity of 4KB128-SAP (C1) produced in** ***P. pastoris*** **for CD22** ^**+**^ **Daudi cells.** Daudi cells were exposed for 72 hours to increasing concentrations of 4KBscFv-SAP (red triangles), seed SAP (light blue squares) or mock supernatant (violet circles) **(A)**. Inhibition of protein synthesis is expressed as percentage of [^3^H]-leucine incorporation compared to untreated control cells. Error bars represent standard deviations from the mean of triplicate samples. **(B)** A competitive inhibition assay was performed by incubating Daudi cells for 72 hours with of 4KB128scFv-SAP at 10^−8^ M in the presence of increasing concentrations of 4KB128 parental monoclonal antibody (filled and open red circles refer to two different batches of 4KB128 MAb). Inhibition of protein synthesis is expressed as percentage of [^3^H]-leucine incorporation compared to untreated control cells. Error bars represent standard deviations from the means of triplicate samples. 4KB128 antibody used alone over the full concentration range was not cytotoxic.

We decided to explore the construct C1 as a prototype for *Pichia pastoris* expression. The untagged C1 construct, however, was difficult to purify, we believe because its isoelectric point was not sufficiently high enough for cation-exchange purification procedure to give the resolution and efficiency needed (data not shown). C1 activity was first assayed on Daudi cells and displayed marked cytotoxicity after 20 hours exposure. C1 cytotoxicity was compared to that of unconjugated seed-extracted saporin (Figure 7A) in a protein synthesis inhibition assay. The recombinant saporin-based scFv fusion showed an IC_50_ of 7 nM, being approximately two orders of magnitude higher than free saporin (Figure 7B) but lower than the conventional (chemical cross-linked) IgG (anti-CD22, 4KB128-SAPORIN) conjugate, reported to be in the order of tens of picomolar [6]. In order to confirm that the C1 activity was mediated via the CD22 target molecule, a competitive inhibition assay was performed by co-incubating Daudi cells for 72 hours with a fixed amount of C1 scFv saporin fusion protein together with increasing concentrations of 4KB128 monoclonal antibody (Figure 7B). An excess of free 4KB128 native antibody competed with the IT for the target antigen and completely abolished C1 cytotoxicity.

As C1 was active and expressed in sufficient amounts, a similar construct termed Construct 4 (C4) was prepared in which a hexahistidine tag was appended to the C-terminus of saporin (Figure 6A, compare C1 and C4) to allow for IMAC affinity purification of the IT.

**Figure 8:**
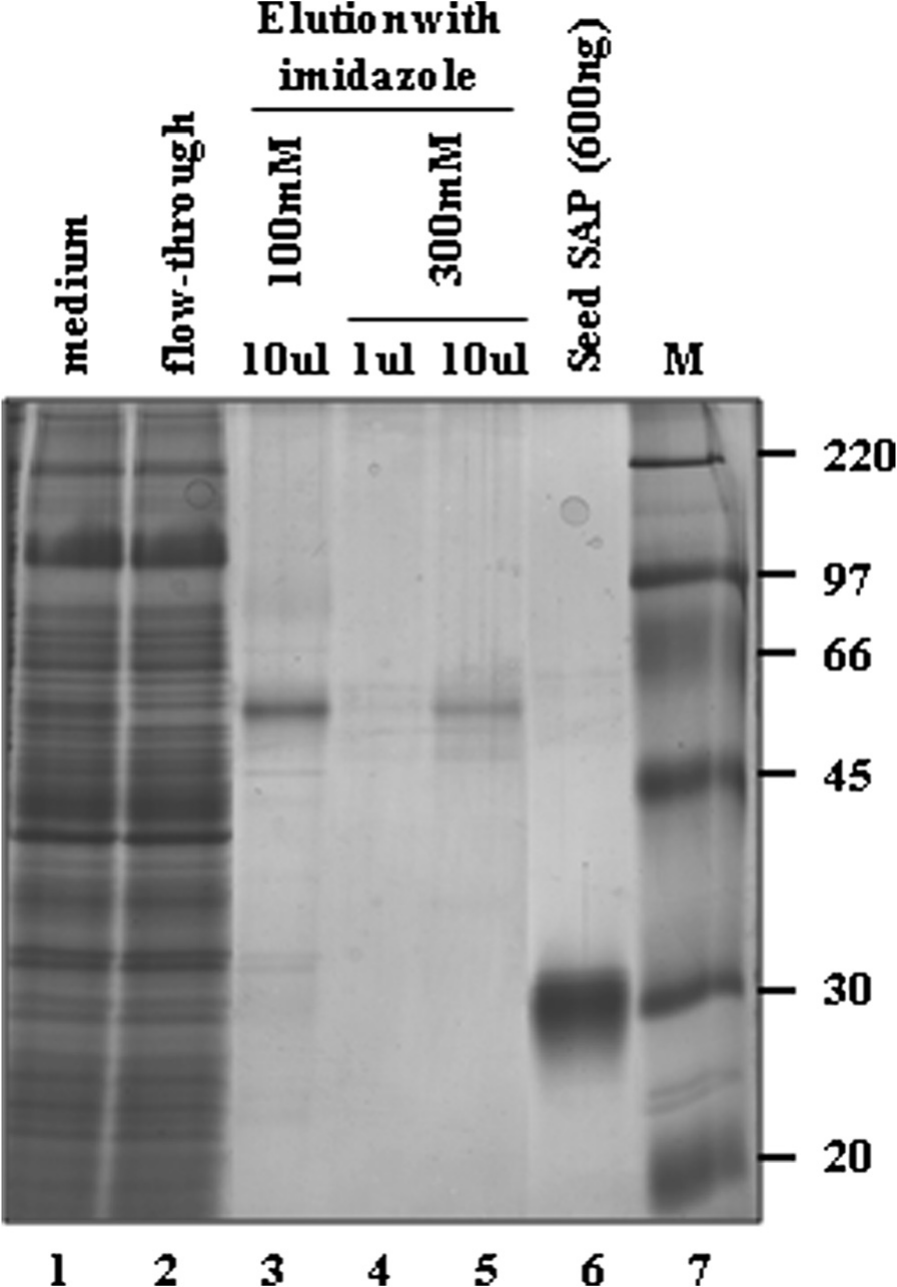
**Characterization of 4KB128-SAP (C4) produced in** ***P. pastoris*** **and purified by IMAC.** Silver staining of purified 4KB128-SAP (C4). MW markers are shown in the far right lane.

C4 purification steps are shown in Figure 8. Unbound material contained a wide range of endogenous proteins, as can be seen in lane 2, but contained virtually no saporin immunoreactivity (data not shown). Elution with 100 mM imidazole was sufficient to detach the majority of the bound C4 scFv-saporin fusion protein with a minor amount eluting at 300 mM imidazole, as evaluated both by the intensity of the single eluted bands in lanes 3 and 5 in the silver-stained gel. This affinity purification procedure allowed for recovery of 30-40% of the induced fusion protein, significantly better than recoveries obtained for the C1 construct purified by ion exchange chromatography.

**Figure 9:**
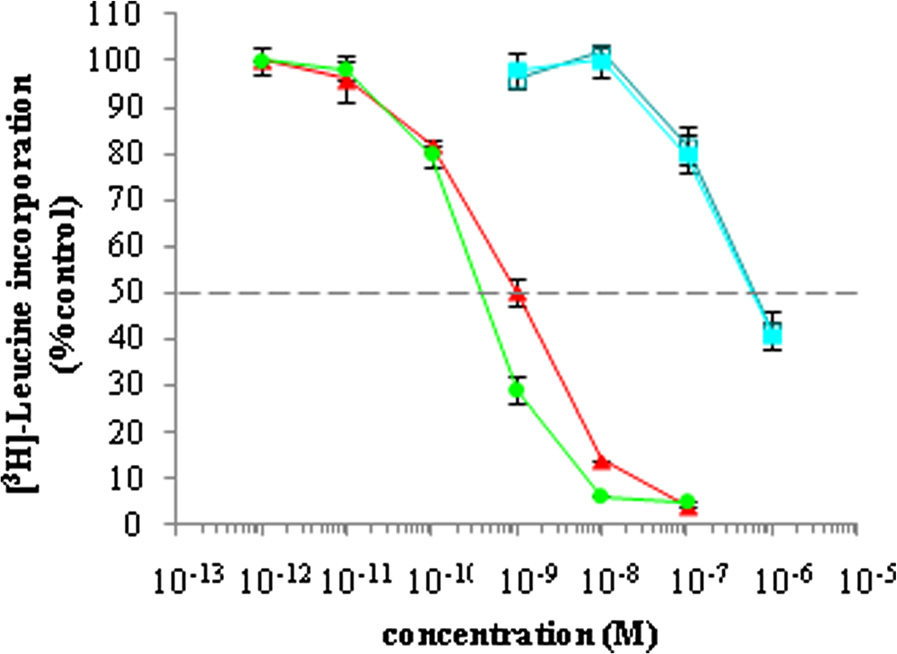
**Protein synthesis inhibition in Daudi cells exposed for 72 hours to increasing concentrations of 4KB-PE40 produced in** ***E. coli*** **(green circles), C4 (4KBopt218L-SAPHis6) (red triangles), rSAP (open blue squares), seed SAP (solid blue squares).** Inhibition of protein synthesis is expressed as percentage of [^3^H]-leucine incorporation compared to untreated control cells. Error bars represent standard deviations from mean of triplicate samples.

Subsequently, the activity of purified C4 construct was assessed on Daudi cells, and was found to be active in the nanomolar range (Figure 9), similar to the cytotoxicity observed for 4KB-PE40 produced in *E. coli*, This indicates that the codon optimization of the scFv and the insertion of the 218 L linker were critical to allow for proper folding, expression and activity of the IT in *Pichia* cells while the His tag did not interfere with its activity contrary to the observations we made with construct 9. The protein synthesis inhibitory activity of the recombinant PE-based scFv fusion was observed to have an IC_50_ of 0.36 nM slightly lower than the 1 nM observed for the C4 anti-CD22 scFv fusion to saporin. We also compared the activity of the above mentioned ITs to that of unconjugated seed-extracted saporin or to recombinant saporin expressed in *P. pastoris*. Notably the latter two displayed identical activity in Daudi cells with an IC_50_ of approximately 0.6 μM. Thus, 4KB218Lopt-SAPHis (C4) is the scFv anti-CD22 fusion to saporin that in our hands performs the best with respect to expression levels and ease and efficiency of purification, with similar cytotoxic activity to construct 1. The activity of the histidine-tagged C4 construct was directly comparable to the untagged C1 construct containing the 218 linker.

### Is bacterial PE efficiently expressed as a fusion with 4KBscFv in *Pichia pastoris*?

**Figure 10:**
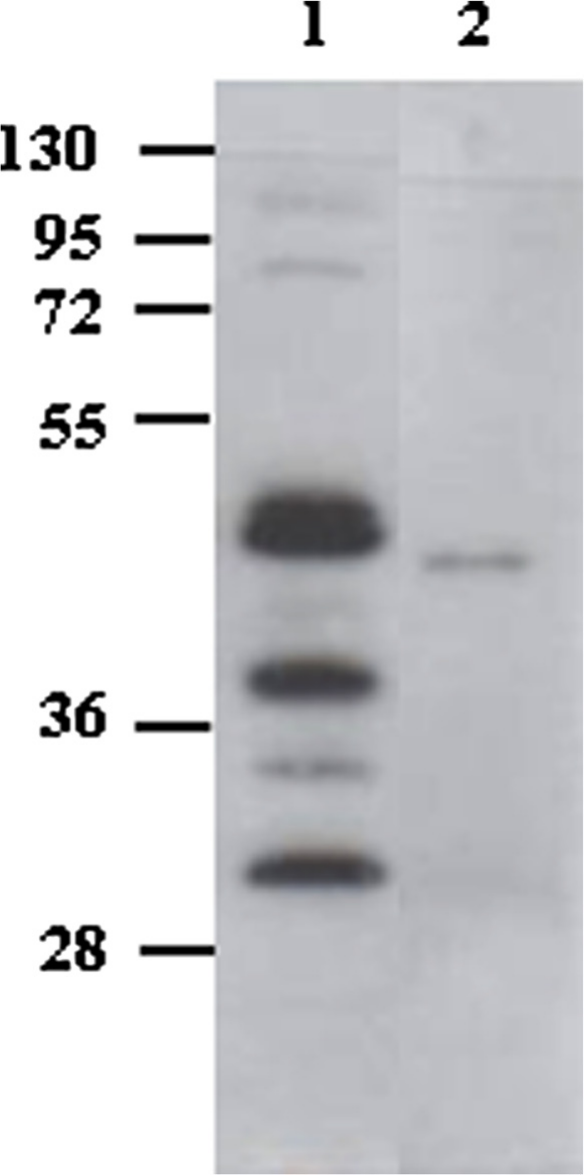
**Expression of 4KB218Lopt-PE40 in** ***P. pastoris*** **.** A sample from a 72-hour medium scale induction of a GS115 clone expressing 4KB218Lopt-PE40 was analyzed by Western blotting with anti-PE serum. Concentrated medium of the induced culture was loaded in *lane 1*; 20 ng of recombinant PE40 expressed in *E. coli* were loaded as a control in *lane 2*.

**Figure 11:**
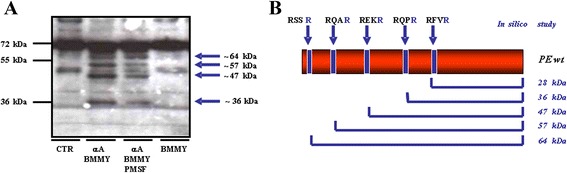
**Cleavage pattern assessment of secreted PE. (A)** Western blot analysis of native PE fragments derived from PE cleaved under different conditions. CTR (control): native PE incubated with PBS; αA BMMY: native PE incubated with BMMY after 48 h of induction of the GS115 mock transformant pPICZαA (αA) clone; αA BMMY PMSF: as αA BMMY but PE was incubated in addition + 1 mM PMSF BMMY: induction medium only*.* **(B)** *In silico* study of identifiable furin-like cleavage sites in the native PE sequence.

Finally, since fusions between antibodies and bacterial toxins have been successfully expressed in *P. pastoris,* as demonstrated by Neville and coworkers for diphtheria toxin [24], we explored the feasibility of expressing PE40 chimeras using this host, in which antibody or other secretory targeting domains might undergo better folding and quality control in the oxidizing environment of the ER lumen. We transformed the eukaryotic host *Pichia pastoris* with the fusion construct 4KB218Lopt-PE40 (Figure 6A) containing the yeast codon-optimized sequences for both the anti-CD22 scFv and the toxin domains. An initial screening of the transformed colonies by Western blot analysis (shown in Figure 10) revealed that no intact polypeptide was secreted into the *P. pastoris* medium and indeed, no band was detectable at the expected molecular mass (70 kDa). A pattern of three bands, presumably corresponding to three possible degradation products with molecular masses of 29, 37 and 42 kDa, respectively were detected. An *in silico* study of the aminoacid sequence of the IT anticipated expressed rIT molecule showed that it contained potential cleavage sites recognized by furin-like enzymes (RXXR) which would be expected to release protein fragments of sizes comparable to those visualized by the Western-blotting. We therefore speculate that proteases present in the yeast medium were responsible for degrading the secreted recombinant 4KB218Lopt-PE40 fusion protein. In this respect several proteases have been described following secretome analysis of *P. pastoris* after methanol induction [32]. To confirm whether this hypothesis is correct, we conducted a study to test if native PE was cleaved by culture medium obtained from *P. pastoris* expressing the recombinant IT following induction by methanol. Native PE was incubated 1 h with: 1) PBS alone as a control, 2) *Pichia* induction 48 h medium, 3) GS115-mock transformed induction medium after a 48 h induction (pPiczαA empty vector) or 4) induction medium containing 1 mM of the serine protease inhibitor PMSF after a 48 h induction. As shown in Figure 11A, native PE incubated with non-inoculated medium remained intact, showing the expected size of PE in the control lane. We can therefore exclude the possibility that proteolytic activity intrinsic to the culture medium was responsible for cleaving the 4KB218Lopt-PE40 recombinant protein. However, when samples of PE were incubated with medium after methanol induction of *P. pastoris* transfectants, four degradation products were observed; these were less intense in the presence of proteases inhibitors. This finding strongly implies that *P. pastoris* secretes proteases into the culture medium that proteolytically cleaves native PE and that the amount of degradation observed was decreased by the addition of protease inhibitors. An *in silico* study of the native PE sequence revealed five putative cleavage sites (Figure 11B): the predicted C-terminal PE fragments have molecular masses similar to those shown by Western-blot analysis. By the mutagenesis of Arg^243^ in the furin cleavage consensus site we could release the 37 kDa peptide fragment. This mutagenesis was performed in the nucleotidic sequence of IL4-PE40, a recombinant immunotoxin available in our laboratory that contains the same codon optimized sequence of PE40 but a different binding domain. The R243A mutation which abolished the furin cleavage site also prevented the degradation of PE C-terminal fragment which could no longer be detected by Western blotting with anti-PE serum (data not shown). Additional studies will be needed to confirm whether after mutagenesis of the potential cleavage sites, a PE-based IT could be expressed that retains intact catalytic activity *in vivo.*

## Conclusions

In the present work we compared a prokaryotic and a eukaryotic expression system used for the production of recombinant immunotoxin molecules based on PE or saporin toxin domains (a flow chart comparing the two expression systems is reported in Additional file 7: Figure S6). Immunotoxins are promising therapeutics for the targeted therapy of leukemia, lymphoma and other malignancies. Targeting CD22 expressed on B-cell tumours with the 4KBscFv fused to either *Pseudomonas* exotoxin A or to the plant toxin saporin would theoretically allow for multiple administrations by switching to a different IT carrying the alternative toxin domain but with the same targeting domain This would be a particularly attractive strategy if a patient develops an antibody response against one of the toxin domain(s) during treatment.

Overall our data demonstrate that we may use a common targeting moiety to deliver toxins of plant or bacterial origin and that the resulting fusion molecules show similar potencies in terms of their protein inhibition capabilities. However, the molecules containing the bacterial toxin are better expressed in the *E. coli* system, whilst the yeast *P. pastoris* is confirmed to be a better host for saporin-based chimaeras in terms of recovery of active products once codon-usage optimization of both the toxin and the targeting scFv domains has been undertaken.

Saporin is a eukaryotic secretory protein and despite its lack of disulphide bonds or N-glycosylation sites, it is a polypeptide that would appear to be better expressed in the environment provided by the endoplasmic reticulum. When saporin is fused to a “non conventional” unfavorable domain, as with the “synthetic” scFv, misfolding may occur and result in greater host toxicity issues, thus reducing expression levels. The reason why codon-usage optimization at least in part, counteracts such an effect by the scFv domain expressed in *Pichia* requires further investigation. The advantage of both the microbial expression platforms used here is that they can both be easily scaled up for industrial production for such therapeutic proteins. Finally, we were able to determine that *P. pastoris* is not a suitable host for the expression of PE-derived fusion proteins because of the potential cleavage sites present in native PE that are recognized by furin-like enzymes secreted by *P. pastoris* into the culture medium.

## Methods

### Materials

All the Materials were of analytical grade.

Recombinant CD22 was purchased from SBH SCIENCES.

4KB128 hybridoma cells were kindly provided by Professor Karen Pulford, University of Oxford and anti-saporin rabbit antiserum was provided by one of our laboratories (DJF/SUF).

The synthetic genes coding for optimized scFv or optimized PE-40 sequence were assembled by Genscript (Piscataway, NJ, USA), based on the available *P. pastoris* coding sequences (CDS) in Biomed Central (64,359 codons with corresponding triplet frequencies, choosing those most frequently represented in highly expressed *P. pastoris* proteins for the construction of the synthetic genes that were subcloned in pUC57 recipient vector, as for the codon-optimized saporin sequence [30] obtaining the pUC57-PE40**opt** construct and 4KB218scFvopt.

The pPICZalpha series of vectors from Invitrogen were used for subcloning the DNA constructs to obtain recipient vectors for expression in GS115 (his4) *Pichia pastoris* strain.

### Plasmid construction for the expressions in *E. coli*

The 4KB128 hybridoma secreting murine IgG directed against human CD22 were cultured under the same conditions used for other cell lines (see below).

Total RNA was extracted using the SV Total RNA Isolation System (Promega, Madison, WI, USA) according to the manufacturer’s instructions. Reverse transcription was performed using M-MLV retrotranscriptase from Invitrogen and a mix of random primers (Invitrogen) to obtain cDNA according to the manufacturer’s instructions.

The sequences coding for the variable domains of heavy (V_H_) and light (V_L_) immunoglobulin chains were amplified by PCR reactions on 1 μg cDNA using a panel of 25 forward and 4 reverse oligonucleotides for each variable domain (25 V_H_ forward primers and 4 J_H_ reverse primers; 25 V_L_ forward primers and 4 J_L_ reverse primers, (see Additional file 1: Table S1). Forward primers were designed based on highly conserved sequences at the 5’-end of DNA fragments for V_H_ and V_L_ domains from several families of murine immunoglobulins; reverse primers were instead inferred from the J regions located at the 3’-end of V_H_ and V_L_ DNA regions. Each forward primer was tested in a PCR reaction that included a mix of the four reverse primers. Once the best forward primer had been thus selected, it was used in four individual PCR reactions, each with a single reverse primer.

The PCR products generated by each of the putative primer pairs were sequenced and compared with sequences present in the Genbank database of variable domains deriving from murine immunoglobulins.

The primer pairs that allowed for a correct amplification of V_H_ and V_L_ genes were then re-designed as modified versions by inserting the suitable restriction sites for the cloning into the recipient vector: *NcoI* and *XhoI* were inserted into the primer for the amplification of the V_H_ chain and *PstI* and *NotI* for the V_L_ chain.

The V_H_ and V_L_ chains were therefore further amplified using the latter pairs of primers, i.e. 4HF, 4HR in the case of amplification of the V_H_ domain and 4KF, 4KR in the case of the V_L_ (Additional file 1: Table S1). The resulting PCR fragments were inserted into a pHEN1 vector derived from a clone obtained from the ETH-2-Gold library and containing a (Gly4Ser)3 linker between the two previously encoded V_H_ and V_L_ regions. The final construct, named 4KBscFv, was amplified with primers 4HF and 4KR (Additional file 1: Table S1) then subcloned into the pET20b(+) expression vector which provided a carboxy-terminal hexahistidine tag for nickel affinity protein purification, in this way we obtained a first construct which we named pET20b(+)4KBscFv(XP). Two point mutations were then inserted into the plasmid pET20b(+)4KBscFv(XP) using the QuickChange Site-Directed Mutagenesis Kit (Stratagene, La Jolla, CA, USA) in order to remove the restriction sites for *PstI* and *XhoI* by respectively using the primer pairs PSTmut1/PSTmut2 and XHOmut1/XHOmut2 (Additional file 1: Table S1). The resulting vector was called pET20b(+)4KB(G_4_S) scFv (Figure 2A).

The sequence of PE40 was amplified from the expression plasmid pHL310 (kindly provided by Prof. Haya Lorberboum-Galski, The Hebrew University, Institute for Medical Research - Israel-Canada, Department of Biochemistry and Molecular Biology, Faculty of Medicine, Jerusalem 91120, Israel) which encodes the IL-2-PE40 fusion protein using PEF and PER primers (Additional file 1: Table S1). The *NotI* cut PCR fragment was inserted at the C-terminus of the 4KBscFv sequence into the pET20b(+) vector cut with the same enzyme to obtain the construct of the immunotoxin 4KB-PE40 (Figure 2B).

To replace the classic (G_4_S)_3_ linker with the longer and more hydrophilic 218 linker, and obtain the 4KB(218)scFv construct in which the heavy and light chains of the variable domains are joined through the 218 linker, two 218 F and 218R oligonucleotides were synthesized (Additional file 1: Table S1). Briefly, the oligonucleotides were mixed with a reaction buffer (100 mM TrisHCl pH 7.5, 200 mM NaCl, 60 mM MgCl_2_), incubated for 2 minutes at 80°C to enable primer annealing and then cooled to room temperature. The primer extension was performed using Klenow fragment (Fermentas) according to the manufacturer’s protocol. The double-stranded fragment was digested with PstI/XhoI and cloned into the pET20b(+)4KBscFv vector to obtain the final construct pET20b(+)4KB(218)scFv.

The sequence of PE40, amplified as described above, was inserted into pET20b(+)4KB(218)scFv at the C-terminus of the scFv sequence to obtain the immunotoxin construct 4KB(218)-PE40his (Figure 2C).

The same cloning strategy was used for construction of the pET20b(+)4KB(218)-SAPhis vector (Figure 2D) amplifying the saporin native sequence from a saporin expression plasmid, as previously described [30] by using the primers SAPF and SAPR (Additional file 1: Table S1).

DH5α-competent bacteria were used for DNA transformation and large-scale preparation, and the putative positive clones were confirmed by sequencing using T7 promoter and T7 terminator primers for pET20b(+) derived constructs. All our DNA sequence analyses were performed by BMR Genomics (Padua, Italy) and primers for DNA amplifications were synthesized by Eurofins MWG Operon.

The *E. coli* strain BL21(DE3)pLysS was transformed with 50 ng of the plasmids described above and grown at 37°C in Luria-Bertani broth containing 100 μg/mL ampicillin. To induce protein expression, 1 mM isopropyl-1-thio-ß-D-galactopyranoside (IPTG) was added to the culture and the induction was carried out for 3 hours at 30°C after which the cell culture was harvested by centrifugation (10000 x g, 10 min).

### Protein purification from bacterial cultures

The bacterial cell pellet was resuspended in 100 ml resuspension buffer (50 mM Na_2_HPO_4_, 0,5 M NaCl pH 7.5). 25 μg/mL DNase, 0,1 mg/mL lysozyme, 1% Triton X-100, 1 mM PMSF, 10 mM MgCl_2_ were added and the solution was sonicated and then centrifuged at 13000 x g for 20 min at 4°C. The precipitated insoluble intracellular fraction was separated and the pellet was dissolved by sonication followed by 2 hours incubation at room temperature in 50 mL of solubilization buffer (8 M urea, 50 mM Na_2_HPO_4_, 0.5 M NaCl pH 7.5).

Solubilized proteins were purified through a 2 mL Ni-Sepharose Fast-Flow resin (GE Healthcare) followed by elution of bound protein with the same solubilization buffer containing 500 mM imidazole.

Refolding of urea-denatured proteins from inclusion bodies was attained by multi-step dialysis in refolding buffer (50 mM TrisHCl, 0.5 M NaCl, 0.4 M L-Arg, pH 7.5) that gradually decreased the concentration of denaturant. The final dialysis step was carried out in PBS (pH 7.2) for 12 hours.

### Rationale for the *Pichia* expression constructs, selection of *pichia* gs115(*his4*) trasformants

A *SfiI-NotI* fragment of a pHEN1 construct containing the 4KB antibody single-chain variable fragment (scFv) was purified and inserted into the *SfiI-NotI*-cut pPICZalphaB recipient vector while a second construct in which the amino acid sequence of this first scFv version was fused to the N-terminus of a saporin yeast codon optimized sequence [30] via an alanine tripeptide linker (encoded within the *NotI* sequence) was also obtained and clone integrity confirmed by DNA sequencing by BMR Genomics (Padua, Italy), that custom performed all the DNA sequence analyses of constructs described herein. A codon-optimized DNA sequence encoding the anti-CD22 scFv was custom synthesized by Genscript company as described previously for the saporin sequence optimization [30] was also used to obtain some of the fusion constructs, following the same cloning strategy.

Electrocompetent GS115 (*his4*) *P. pastoris* cells were prepared according to protocols from Invitrogen. A best expresser strain GS115 (his4) able to support PA63-saporin expression was used as control in some inductions. A Bio-Rad Gene pulser apparatus (Bio-Rad, Milan, Italy) was used for electroporation of linearized DNA constructs for genomic integration. DNAs were carefully quantitated in ethidium-bromide-stained agarose gels, and equivalent amounts of DNA (5–10 μg) resuspended in sterile water were used for each electroporation cuvette. Linearized empty pPICZalpha vectors were always used as control for the mock-transformant cells. Then, either 200 or 600 μl of transformed cells were plated for selection on YPD [1% (w/v) yeast extract, 2% (w/v) peptone or tryptone, and 2% (w/v) dextrose] plates containing 18.2% sorbitol (YPDS) in the presence of 1.5% (w/v) agar and 50 μg/mL Zeocin (Invitrogen). Colonies started to appear after 3–4 days incubation at 30°C, and randomly selected colonies were restreaked onto YPDS-zeocin plates. In the case of non optimised scFv fusion constructs only few colonies could be selected and almost no induction or very little expression of the fusion construct could be observed in one or two clones.

At least 10 different fusion constructs have been designed, produced and separately introduced into *Pichia* GS115(*his 4*) cells, using as a starting point the yeast codon optimised saporin (SAPopt) sequence which has been fused to alternate versions of the anti-CD22 single chain variable fragments that were collectively termed 4KB. For further details please refer to Figure 6A in which clone identifying numbers refer to the (best) reference clone(s) obtained and further analyzed for each transformation set. AAA in aminoacid one letter code refers to the encoded Alanine linker joining the two variable single chain domains to the toxin domain. Basic fusion construct configuration generally included the Preproalpha factor domain which was always used as a common yeast secretory domain, in all our constructs (not shown in Figure 6) optimized saporin followed by AAA linker, except for constructs labeled 7, 8 that started with the heavy chain variable (V_H_) antibody domain immediately following the Preproalpha factor domain and construct 9 which has saporin domain connected via a G_4_S linker to the scFv optimized 4KB domain.

We also introduced and investigated the effects of a change in linker sequence between V_H_ and V_L_, leading to what we term “218 L derived constructs”. Two alternate construction options were explored, by inserting instead of the classic (G_4_S)_3_ linker between the V_H_ and V_L_ antibody domains, a longer and more hydrophilic 218 linker. We also checked for the expression of fusions with an hexahistidine tag placed either at the C-terminus of the fusion toxin or at its N-terminus, in the latter case the 6xHistag was placed just beyond an endoprotease Kex-2 like site that should be recognised allowing for removal of the transient alpha factor secretory domain within the Golgi complex. Overall, only two construct(s) gave us a successful clones, construct 1 and construct 4 that are quite similar, because they both contain the 218 linker between V_H_ and V_L_ of the codon yeast optimized 4KBopt sequence, differing only for the presence of an histidine tag at the saporin C terminus in clone 4.

After obtaining the pUC57-PE40**opt** construct from Genscript, the PE40 optimized sequence, including the C-terminal hexahistidine tag, was amplified by PCR on pUC57-PE40opt with optPE40 and optPE6his primers (Additional file 1: Table S1) and ligated in the *NotI-XbaI*-cut pPicZalphaB-4KBopt218L; the final construct was named 4KBopt(218)-PE40his (Figure 6A).

### *Pichia* expression screening procedures and large scale induction conditions

Screening conditions were either small-scale inductions of *Pichia* independent clones after being restreaked onto YPDS-zeocin plates. Single colonies were picked into 5–10 mL broth and at 2OD/mL these were either non-induced or induced for 48 h with 0.5% Methanol in BMMY. Equivalent samples of non-induced (NI) or methanol-induced (I) media were loaded, subjected to a SDS-PAGE and Western-blot analysis using anti-saporin serum, as shown in Figure 6B.

As negative control, an induced mock-transformant clone was also loaded in same amounts, to confirm no reactivity was present, whereas as a positive control of a small-scale induction of the model IT antiPA63-SAP [30] was also loaded, to confirm the expected size and to roughly compare expression levels of the fusion polypeptides under small-scale induction conditions, which were also compared to standard seed saporin for protein quantitation’s.

Among the 20 clones picked and induced, we then selected a “best expresser” clone showing almost no immunoreactivity in the NI-condition but when induced showing an immune-reactive band of the expected size for a saporin-scFv fusion (around 55 KDa) co-migrating with the model control scFv fusion. In some experiments we noticed the presence of faint reactive bands migrating at the size of saporin in some of the induced media.

Larger scale inductions in 400 mL of the best expresser clones were performed as previously described (See Additional file 2: Figure S1). In some cases when several hundred clones were obtained after *Pichia* transformation, inductions of colony lifts were performed as described in detail in [30] and shown here in Additional files 3 and 4: Figures S2-S3.

### Protein purifications from *P. pastoris* culture

Clone 1 construct was purified by a cation Exchange using Resource S essentially as described [21], with only low amounts of fusion protein recovered. The clone 4 construct (4KBopt218L-SAPHis6) and the 4KBopt218L-PE40 supernatants were loaded onto Proteus IMAC kit (AbD Serotec, Oxford, UK), after concentration of medium essentially following the manufacturer’s instructions, except that 25 mM imidazole was used in the binding buffer during sample loading and 3 washes with 50 mM imidazole in the wash buffer were performed before elution in the presence of increasing concentrations of imidazole (150, 300, and 500 mM). A first peak eluted with 150 mM imidazole. Eluates were exchanged against PBS (pH 7.6) by dialysis and concentrated to 1 mL using Vivaspin 10,000 cutoff concentrators (Vivascience; Sartorius Stedim Biotech) following centrifugation at 5000 g. Samples were analyzed by SDS-PAGE and subjected to silver staining or Western blotting, using SAP-S as a standard.

### SDS-PAGE, Western blot and Coomassie-blue staining

SDS-PAGE was performed on 12% polyacrylamide gels. For Western blot analysis, proteins transferred onto PVDF membranes (Millipore) were probed with a mouse anti-His IgG antibody (GE Healthcare), a rabbit anti-Pseudomonas Exotoxin A serum (Sigma-Aldrich) or a rabbit anti-saporin anti-serum.

### Cell lines

The biological assays were performed on two human lines of B lymphocytes derived from Burkitt’s lymphoma and expressing CD22 antigen (Daudi or Ramos cell line) and two CD22-negative T-lymphoblastoid cell lines (HSB-2 and H9). Cells were cultured in RPMI 1640 medium (with 40 mg/L folic acid, 2 g/L NaHCO3) (Biochromag) supplemented with 10% FCS, 2 mM L-Glutamine and antibiotics (100 U/mL penicilline and 100 μg/mL streptomycine-sulphate). Daudi cells were grown in flasks at 37°C in a 5% CO_2_ humidified atmosphere.

### Binding properties of the fusion proteins to CD22 antigen

The binding characteristics of 4KB128 monoclonal antibody and of the 4KB128-derived scFv and rITs for CD22 antigen was assessed using the CD22-positive cell line Daudi by incubating 3 × 10^5^ cells with increasing concentration of purified mAb 4KB128 or scFv (ranging from 10^−11^ M to 10^−5^ M) or rITs (from 10^−9^ M to 10^−5^ M). Cells were analyzed for fluorescence on a FACS Caliber flow-cytometer after a staining with anti-His antibody and a second incubation with a goat anti mouse-FITC antibody (Beckman Coulter).

In the competition assay 3 × 10^5^ Daudi cells were preincubated on ice for 20 minutes with increasing amounts of the purified anti-CD22 mAb 4KB128. Five μg of the scFv (100 μl) was then added and incubation continued for one hour on ice. Cells were then stained with anti-His6-FITC (Miltenyibiotec) and analyzed as described above. The inhibition of scFv binding was evaluated by reference to the maximal MFI without competing 4KB128 antibody.

### Stability and internalization in target cells

The stability of the anti-CD22 mAb and of the derived scFv was evaluated by incubation of the antibodies at 37°C for the same times as in the internalization experiment (see below). The two antibodies were diluted at concentrations of 0.5 μg/mL (mAb) and 10 μg/mL (scFv) and incubated for up to 60 minutes at 37°C in a water bath. At each time point the corresponding tube was transferred in ice and analysed by flow cytometry as described above.

Internalization of anti-CD22 mAb and scFv was assayed on target cell lines Ramos and Daudi. 3 × 10^5^ cells were incubated on ice with 3 μg of scFv or 1 μg of mAb in a final volume of 100 μl for 1 hour. After two washes cells were maintained at 37°C in water bath for 0, 2, 5, 10, 20 or 60 minutes. Next, the scFv or mAb retained on the surface of the cells were detected with anti-His antibody followed by anti mouse-FITC for the scFv or with the anti mouse-FITC only for the mAb.

### Cytotoxicity assays

Serial dilutions of rITs ranging from 10^−8^ to 10^−13^ M were added to 2.5 × 10^4^ cells seeded in 96-well plate and maintained in leucine-free RPMI-1640 medium with 2% fetal calf serum (FCS). After incubation for 48 hours at 37°C, 0.2 μCi of [^14^C]-leucine or 0.5 μCi of [^3^H]-leucine were added. Incorporated radioactive leucine was measured on a beta counter.

The specific inhibition of 4KB-PE40 IT activity was determined in a competition assay in which Daudi cells were seeded 2.5 × 10^4^ in a 96-well plate, and incubated with increasing concentrations of 4KB-PE40 in the presence or absence of a fixed concentration of the CD22 mAb 4KB128 (10^−6^ M). After an incubation period of 48 hours at 37°C, [^14^C]-leucine was added and the incorporated radioactivity was measured as described above.

## Additional files

Additional file 1: Table S1. Oligonucleotide sequences used to generate expression plasmids. Additional file 2: Figure S1. Example of medium and small scale induction procedures. Additional file 3: Figure S2. Screening for constructs 7, 8 on plate. Additional file 4: Figure S3. Screening for construct 9-induced clones on plate. Additional file 5: Figure S4. Comparison of secretion yields of *Pichia pastoris* clones deriving from constructs 6–9. Additional file 6: Figure S5. N-terminal histidine tagged fusions (construct C6, see also Figure 6) were not recognized be the anti-tag antibody. Additional file 7: Figure S6. Flow chart representation comparing the two expression systems tested.

## Abbreviations

IT: Immunotoxin
MFI: Mean fluorescence intensity
PEA: Pseudomonas exotoxin A
PE40: Truncated version of Pseudomonas exotoxin A
scFv: Single-chain variable fragment
