# Supplementary material for: Systematic comparison of single-chain Fv antibody-fusion toxin constructs containing Pseudomonas Exotoxin A or saporin produced in different microbial expression systems
Source: Microb Cell Fact. 2015 Feb 13;14:19. doi: 10.1186/s12934-015-0202-z (PMC4338634; doi:10.1186/s12934-015-0202-z)
Supplement: Additional file 1: Table S1. — Oligonucleotide sequences used to generate expression plasmids. [file 12934_2015_202_MOESM1_ESM.doc]

**Table S1. Oligonucleotide sequences used to generate expression plasmids**

| **Name** | **Primer** | **Oligonucleotide sequence** | **Restriction site** |
| --- | --- | --- | --- |
| 4HF | Forward | 5’-GTCCTCGCAACTGCGGCCCAGCCGGCCATGGCCGAGTGAAGCTGATGG  AATCTGG-3’ | *Nco*I |
| 4HR | Reverse | 5’-GAGTCATTCTGCTCTCGAGACGGTGACTGAGG-3’ | *Xho*I |
| 4KF | Forward | 5’-CATGACCACACTGCAGTCGACATTGTTCTCACCCAGTCTCC-3’ | *Pst*I |
| 4KR | Reverse | 5’-GAGTCATTCTGCGGCCGCCCGTTTTATTTCCAGCTTGGTCCC-3’ | *Not*I |
| XHOmut1 | Forward | 5’-AAAACGGGCGGCCGCACTTGAGCACCACCACCACCAC-3’ |  |
| XHOmut2 | Reverse | 5’-GTGGTGGTGGTGGTGCTCAAGTGCGGCCGCCCGTTTT-3’ |  |
| PSTmut1 | Forward | 5’-TGACACCACGATGCCTGCTGCAATGGCAACAACGTTG-3’ |  |
| PSTmut2 | Reverse | 5’-CAACGTTGTTGCCATTGCAGCAGGCATCGTGGTGTCA-3’ |  |
| 218F | Forward | 5’-ATCTCGAGTGGCTCCACCAGCGGCAGCGGCAAGCCAGGCTCCGGCGAA-3’ | *Xho*I |
| 218R | Reverse | 5’-AATTCTGCAGAGCCTTTGGTGCTGCCTTCGCCGGAGCCTGGC-3 ’ | *Pst*I |
| PEF | Forward | 5’- TATAGCGGCCGCTTCCGGAGGTCCCGAG-3’ | *Not*I |
| PER | Reverse | 5’-TATAGCGGCCGCCTTCAGGTCCTCGCGCGG-3’ | *Not*I |
| SAPF | Forward | 5’-TATAGCGGCCGCTTCCGGAGGTGTCACATCAATC-3’ | *Not*I |
| SAPR | Reverse | 5’-TATAGCGGCCGCCTTTGGTTTGCCCAA-3’. | *Not*I |
| optPE40 | Forward | 5’-TTCCGCGGCCGCAGGTGGTCCAGAA-3’ | *Not*I |
| optPE6his | Reverse | 5’-TTCTAGATTAATGATGATGATGATGATG-3’ | *Xba*I |
