# Supplementary figures and images for: Systematic comparison of single-chain Fv antibody-fusion toxin constructs containing Pseudomonas Exotoxin A or saporin produced in different microbial expression systems
Source: Microb Cell Fact. 2015 Feb 13;14:19. doi: 10.1186/s12934-015-0202-z (PMC4338634; doi:10.1186/s12934-015-0202-z)

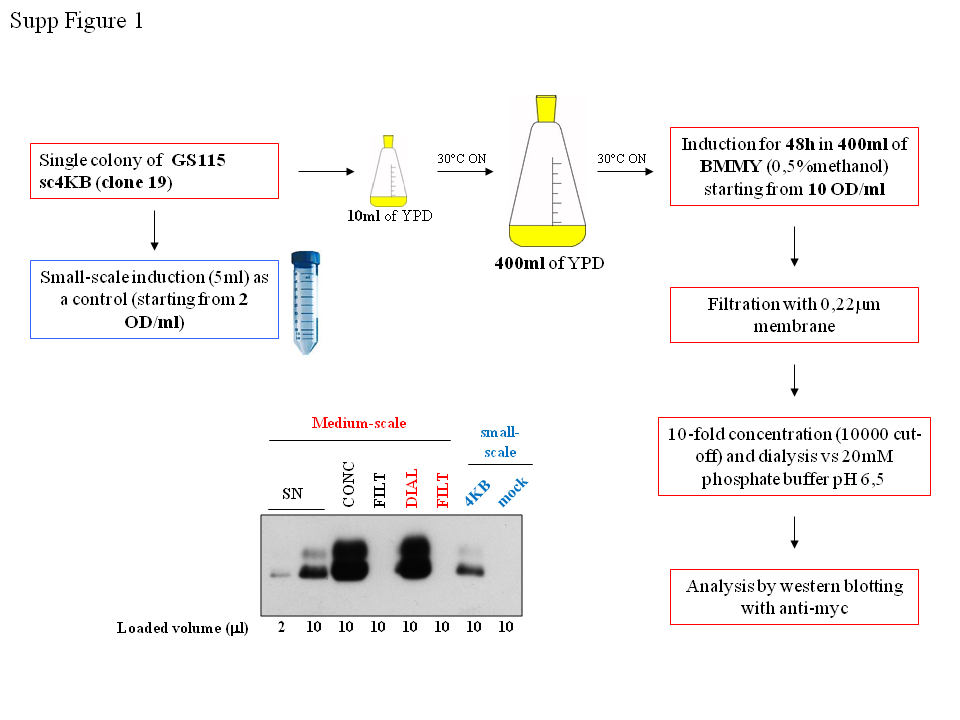

Supplement: Additional file 2: Figure S1. — Example of medium and small scale induction procedures. [file 12934_2015_202_MOESM2_ESM.tiff]

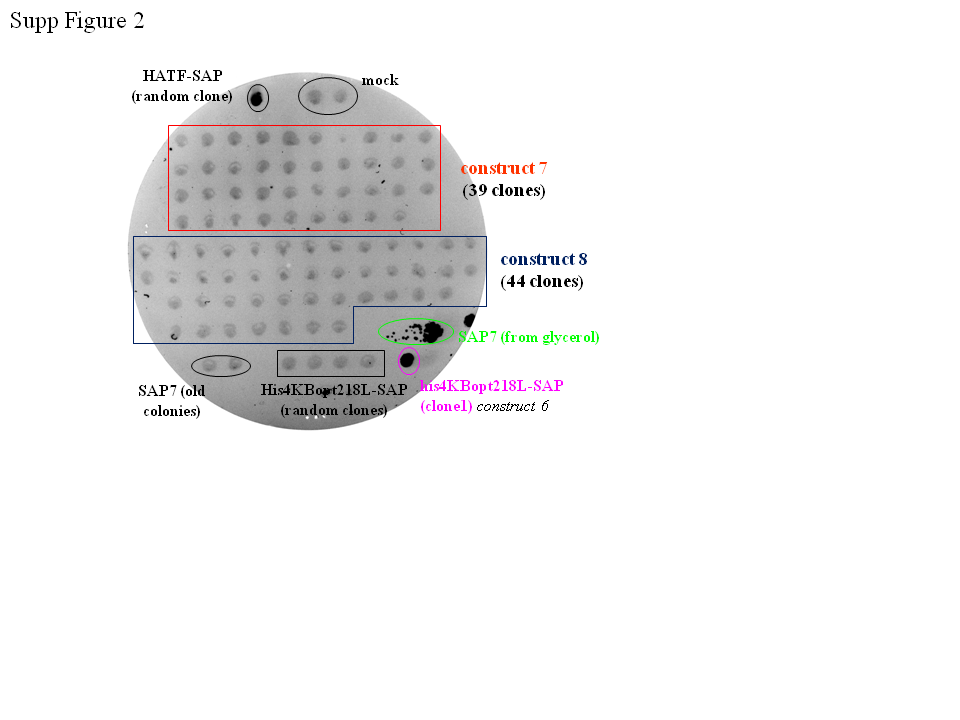

Supplement: Additional file 3: Figure S2. — Screening for constructs 7, 8 on plate. [file 12934_2015_202_MOESM3_ESM.tiff]

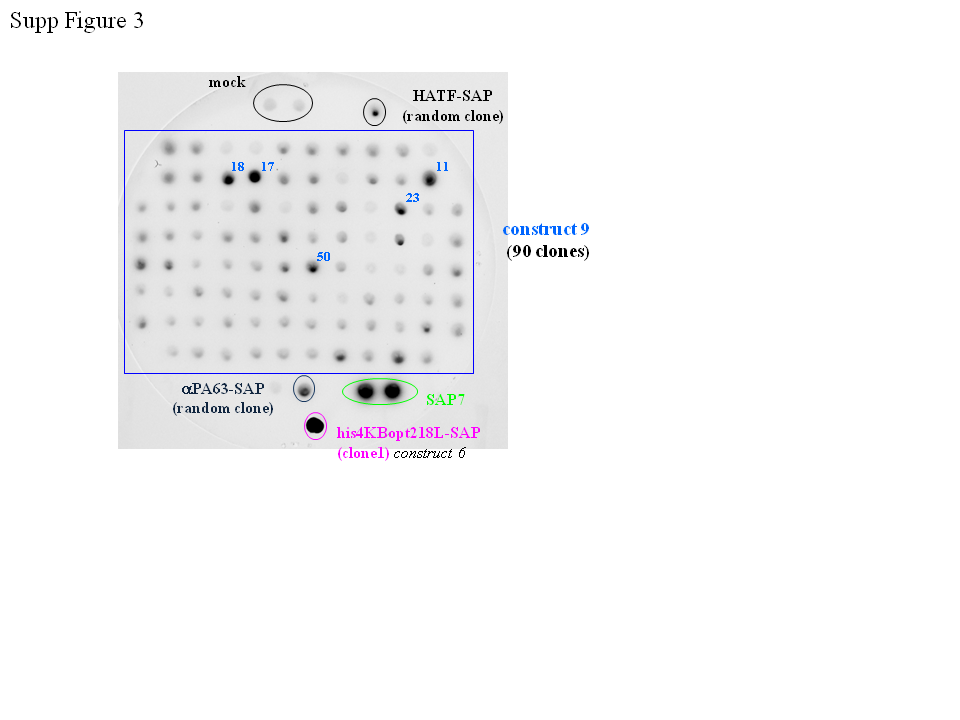

Supplement: Additional file 4: Figure S3. — Screening for construct 9-induced clones on plate. [file 12934_2015_202_MOESM4_ESM.tiff]

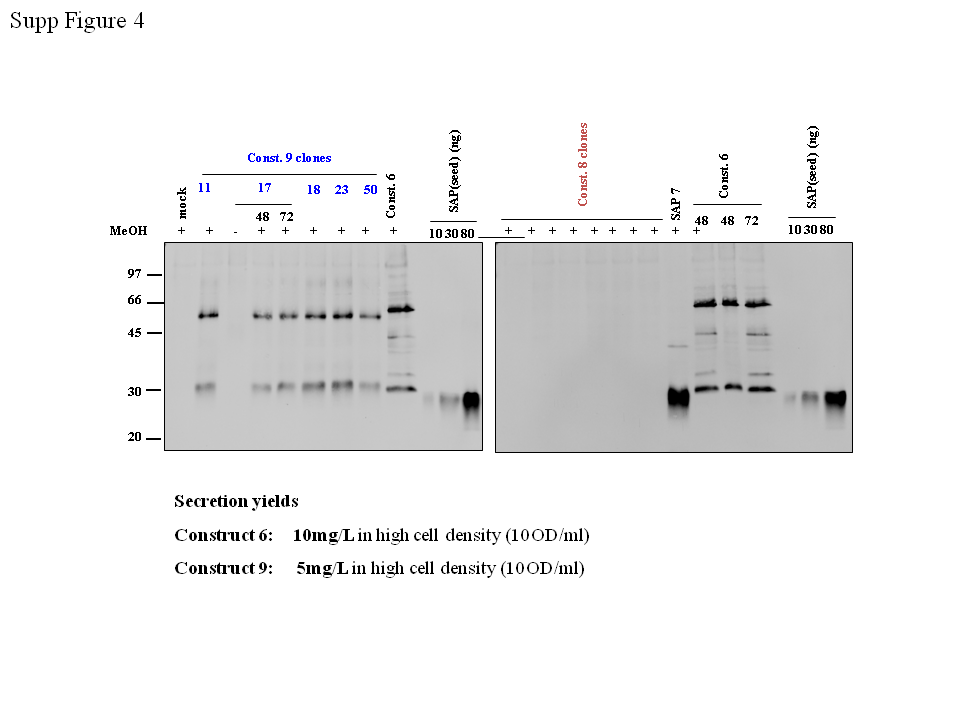

Supplement: Additional file 5: Figure S4. — Comparison of secretion yields of Pichia pastoris clones deriving from constructs 6–9. [file 12934_2015_202_MOESM5_ESM.tiff]

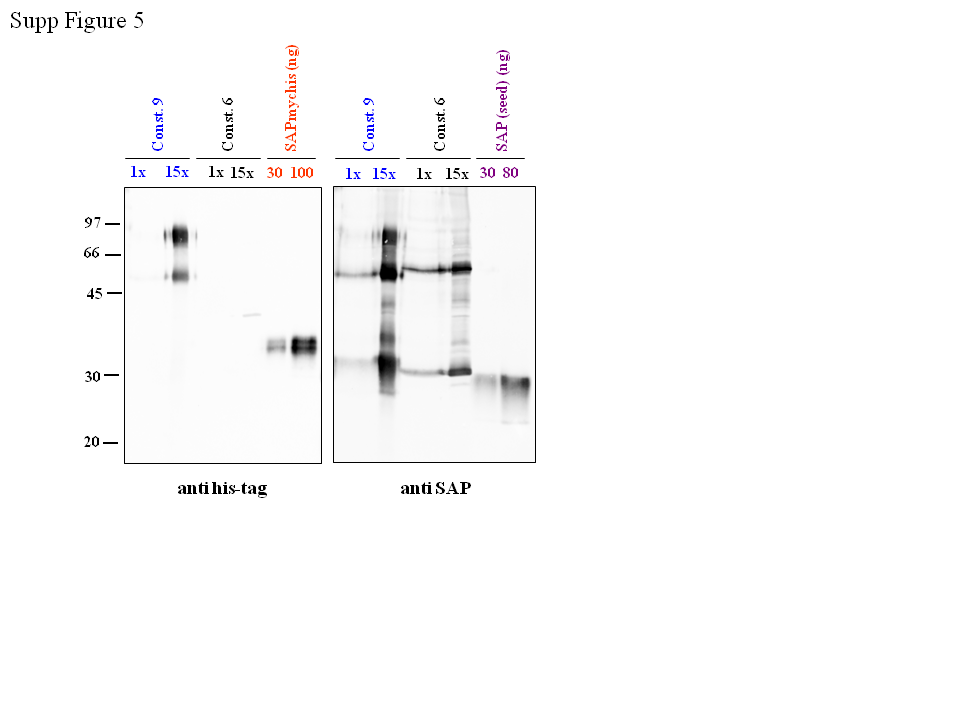

Supplement: Additional file 6: Figure S5. — N-terminal histidine tagged fusions (construct C6, see also Figure 6) were not recognized be the anti-tag antibody. [file 12934_2015_202_MOESM6_ESM.tiff]

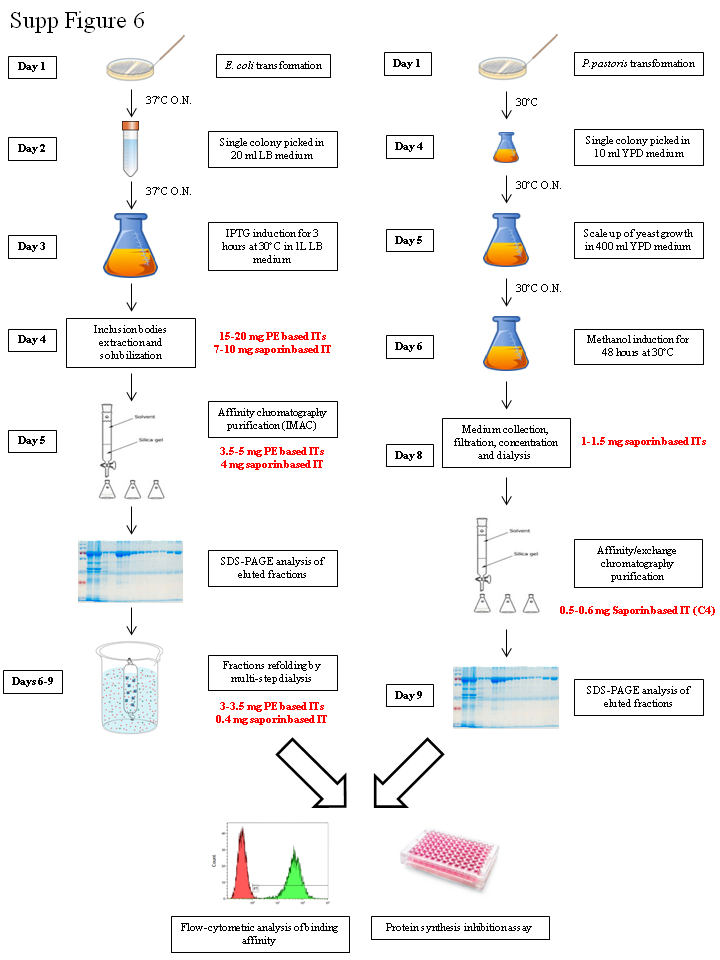

Supplement: Additional file 7: Figure S6. — Flow chart representation comparing the two expression systems tested. [file 12934_2015_202_MOESM7_ESM.tiff]
